# Protective Effects of Astaxanthin on Thioacetamide-Induced Hepatopancreatic Damage in *Procambarus clarkii*: Insights from Biochemical, Histological, and Metabolomic Analyses

**DOI:** 10.3390/ani15111537

**Published:** 2025-05-24

**Authors:** Jiawen He, Jian Ju, Qingliang Jiang, Haiyong Zhao, Yingying Zhang, Hui Yang

**Affiliations:** 1 College of Bioscience and Biotechnology, Yangzhou University, 48 Wenhui Road, Yangzhou 225009, China; jiawen_he@163.com; 2 College of Animal Science and Technology, Yangzhou University, Yangzhou 225009, China; 13328143718@163.com (J.J.); jql_050914@163.com (Q.J.); zhangyingying@yzu.edu.cn (Y.Z.); 3 Chuzhou Heyuxing Biotechnology Co., Ltd., Chuzhou 239000, China; 13641654190@163.com

**Keywords:** *P. clarkii*, astaxanthin product, oxidative stress, hepatopancreas injury

## Abstract

The hepatopancreas of *Procambarus clarkii* plays a crucial role in metabolism and immune response, encompassing vital physiological functions. In our study, we established a hepatopancreatic injury model using thioacetamide (TAA) and evaluated the therapeutic potential of a commercial astaxanthin-based product (AST-product) on hepatopancreatic health. The experimental framework included a control group (Con), an injury model group (M), and a treatment group (T), enabling a comprehensive analysis of the effects of treatments on hepatopancreatic biochemical markers, tissue architecture, gene expression, and metabolic pathways. The biochemical results indicated significant oxidative damage and fibrosis in the M group post-TAA treatment, evidenced by increased malondialdehyde (MDA) levels, decreased superoxide dismutase (SOD) activity, and the disruption of tubular structures. Conversely, treatment with the AST-product significantly reduced MDA levels and ameliorated oxidative stress. Histological evaluations using hematoxylin and eosin (HE) and Sirius Red staining confirmed that the AST-product preserved tubular integrity and inhibited fibrosis progression. Metabolomic profiling revealed that the AST-product modulated key metabolic pathways, including arginine and proline metabolism, porphyrin metabolism, and nucleotide metabolism, which are critical for maintaining energy supply and antioxidative capabilities. This modulation mitigated the TAA-induced oxidative damage. Moreover, qPCR analysis demonstrated that the AST-product downregulated the pro-apoptotic gene CASP2, upregulated the energy metabolism-related gene NDUFA7, and enhanced the expression of the immune-related gene lysozyme, thereby boosting pathogen resistance. These findings elucidate the robust protective effects of the AST-product on hepatopancreatic health in *P. clarkii*, highlighting its potential to reduce oxidative stress, inhibit fibrosis, and enhance immune responses. This study provides a novel strategy for improving *P. clarkii* health in aquaculture and contributes valuable insights into hepatopancreatic protection and disease prevention in shrimp farming.

## Simple Summary

Crayfish are a commercially important species in aquaculture, but their health and growth can be compromised by liver and pancreas damage which is often triggered by environmental stress and chemical exposure. These organs are vital for digestion, metabolism, and immunity. In this study, we established a disease model in crayfish by using thioacetamide, a chemical that induces liver damage, to simulate realistic farming stress. We then investigated whether a natural astaxanthin-based feed additive could protect crayfish against this damage. Crayfish that were fed the supplemented diet showed clear improvements in tissue structure, lower levels of oxidative stress, and reduced signs of fibrosis. In addition, their antioxidant activity, immune responses, and energy metabolism were enhanced. These protective effects were supported by both tissue analysis and molecular data. Our findings suggest that astaxanthin-based supplements may be a promising strategy for improving organ health and disease resistance in crayfish. This approach could help reduce losses in aquaculture caused by stress-related diseases, contributing to more sustainable and healthier farming practices.

## 1. Introduction

*Procambarus clarkii* (Girard, 1852), a globally distributed freshwater crustacean, demonstrates remarkable adaptability and has become an integral part of China’s aquaculture industry due to its high yield and significant economic value [1,2]. Among its physiological systems, the hepatopancreas is a critical organ, playing pivotal roles in metabolism, growth, and development, essential for maintaining overall physiological health [3]. However, the hepatopancreas is susceptible to a variety of biotic and abiotic stressors such as heavy metals, plastic particles, pathogenic bacteria, and algae, which can inflict significant damage, impairing tissue structure, antioxidant enzyme activities, and gene expression [4,5,6,7]. Such damage can trigger severe pathological reactions, potentially leading to extensive mortality and substantial economic losses.

Recent advances in the field of hepatopancreatic protection have identified both chemical and biological agents as effective in maintaining the health of this organ in shrimp. For instance, the inclusion of 80–160 mg/kg of vitamin E in the diet has been demonstrated to promote growth performance and hepatopancreatic function in *Macrobrachium japonicus* [8]. Additionally, *Lactobacillus plantarum* Ep-M17 has been found to enhance immune responses and metabolic activities in the hepatopancreas of *Penaeus vannamei*, thereby improving disease resistance [9]. Similarly, hemocyanin has been reported to preserve mitochondrial integrity, regulate energy metabolism, and control reactive oxygen species production, which in turn influences the hepatopancreatic microbiota in *P. vannamei* [10]. Moreover, bacteriophage therapy has been explored as a novel method to prevent hepatopancreatic necrosis disease in *P. vannamei* [11]. Several recent dietary strategies have also shown promising effects on hepatopancreatic health. Dietary supplementation with a medium-chain fatty acid product significantly improves the structural integrity and immune function of the hepatopancreas in white shrimp [12]. Similarly, the dietary inclusion of the phytobiotic-based additive Sanacore^®^ GM (Adisseo, Dendermonde, Belgium) significantly improves hepatopancreas health in Pacific white shrimp by enhancing immune-related enzyme activities and preserving tissue architecture [13]. Supplementation with myoinositol effectively alleviates hepatopancreatic damage and oxidative stress induced by oxidized fish oil in *L. vannamei*, thereby improving antioxidant capacity, tissue structure, and metabolic enzyme function [14]. Fermentation products of *Bacillus velezensis* T23 have been shown to improve hepatopancreas health in *L. vannamei* by increasing antioxidant enzyme activities and reducing inflammation and tissue injury [15]. In addition, lysophospholipid supplementation in low-fishmeal diets significantly enhances hepatopancreas health in *L. vannamei* by boosting antioxidant and digestive enzyme activities, modulating lipid metabolism, and preserving histological structure [16]. These findings underscore the importance of identifying safe and effective functional additives to support hepatopancreatic health in crustaceans under stress. Among the various candidates, astaxanthin has emerged as a promising compound due to its broad physiological benefits and antioxidative properties.

Astaxanthin, a carotenoid that can be obtained from various natural sources including microalgae such as *Haematococcus pluvialis*, yeast (*Phaffia rhodozyma*), and marine animals, as well as through synthetic processes, has garnered attention for its biological functions and commercial applications [17]. Research indicates that dietary supplementation of 200 mg/kg of astaxanthin can significantly enhance the growth and immune response of *P. clarkii*, effectively reducing mortality rates [18]. Further studies have shown that appropriate levels of dietary astaxanthin improve the activities of antioxidant enzymes, thereby mitigating stress responses in *P. clarkii* [19]. Additionally, adding 9–12 mg/L of astaxanthin to feed can prevent the bioaccumulation of microalgal toxins in the hepatopancreas and ovaries, enhancing survival and growth rates [20]. Astaxanthin exhibits a diverse array of physiological benefits in aquaculture species, including potent antioxidant activity, immunomodulatory effects, the promotion of growth, the enhancement of reproductive capacity, and the improvement of body pigmentation [21]. At the molecular level, astaxanthin mediates its effects through multiple signaling pathways: it activates the nuclear factor erythroid 2-related factor 2 (Nrf2) antioxidant defense system, modulates the phosphatidylinositol 3-kinase/protein kinase B (PI3K/Akt) and AMP-activated protein kinase (AMPK) pathways, and regulates the expression of genes associated with the oxidative stress response, immune function, and energy metabolism [22,23]. These multifaceted properties not only enhance the overall physiological resilience of aquatic animals but also underscore astaxanthin’s potential as a therapeutic agent for alleviating chemically induced hepatopancreatic fibrosis. However, astaxanthin’s chemical instability under environmental stressors such as high temperatures, acidic pH, and light exposure presents a major limitation to its practical use. To overcome this, the nanocapsulation of astaxanthin-rich lipid extracts has been proposed as a promising strategy to improve its stability and bioavailability [24].

In this study, we established a hepatopancreatic fibrosis model in *P. clarkii* using thioacetamide (TAA) [25], followed by treatment with a highly stable and bioavailable astaxanthin-based commercial product (Yangzhou Fish Doctor Fisheries Science and Technology Co., Ltd., Yangzhou, China). We hypothesized that dietary supplementation with a stable astaxanthin product can alleviate TAA-induced hepatopancreatic damage in *P. clarkii* by reducing oxidative stress, preserving tissue structure, and modulating key metabolic and genetic pathways. We evaluated the therapeutic effects of astaxanthin by assessing enzyme activities related to hepatopancreatic function, conducting histological examinations, performing metabolomic analyses, and utilizing quantitative real-time PCR (qPCR). This research aims to delineate the protective mechanisms of astaxanthin on the hepatopancreas of *P. clarkii*, offering novel theoretical insights and practical approaches for sustainable health management and disease prevention in crayfish aquaculture.

## 2. Materials and Methods

### 2.1. Experimental Animals

*P. clarkii* specimens were sourced from Qianjiang, Hubei Province, China. The selection criteria included intact appendages, high vitality, and uniformity in size, with an average body weight of 10.5 ± 0.45 g and an average body length of 8.5 ± 0.6 cm. Prior to experimentation, the crayfish were acclimated for seven days in a circular PVC plastic culture tank (1.0 m radius, 1.5 m height) with a constant water depth of 1.0 m. The water, aerated tap water, was pre-aerated for 72 h and maintained at a temperature of 25 ± 1 °C with continuous aeration to ensure adequate dissolved oxygen levels. The water quality was preserved by replacing one-third of the volume every three days.

### 2.2. Experimental Design

The astaxanthin-based commercial product (AST-product) was purchased from Yangzhou Fish Doctor Fisheries Science and Technology Co., Ltd. The product contained astaxanthin ≥ 10% (HPLC-purified), derived from *H. pluvialis*, and was stabilized with antioxidants and encapsulated to improve bioavailability. Prior to the experiment, all crayfish were fasted for 12 h. Each specimen received an intramuscular injection of 100 μL of TAA in saline (Macklin, Shanghai, China) to deliver a final dosage of 0.8 mg/g of their body weight. The control group received a 100 μL saline injection instead. After a 24 h acclimation period, the crayfish were allocated into three groups: control (Con), liver injury model (M), and treatment (T), with each group consisting of three replicates of 20 crayfish each, totaling 180 specimens. The AST-product powder was dissolved in distilled water, mixed with feed, homogenized on an orbital shaker for 30 min, and then dried in an oven. The astaxanthin to feed weight ratio was maintained at 1 g/100 g. Feeding occurred twice daily at 09:00 and 16:00, with each meal constituting 2% of the total crayfish body weight. The Con and M groups received standard feed, whereas the T group was given astaxanthin-enriched feed. This feeding regimen continued for 14 days with tri-weekly water replacements to ensure water quality. At the beginning and end of the experimental period, 10 crayfish per replicate from each group were selected for hemolymph extraction to prepare plasma, and the hepatopancreatic tissue was harvested. For analyses, tissue from five crayfish per group was frozen at −80 °C to measure various markers of hepatopancreatic injury and oxidative stress, as well as for metabolomic studies. Tissues from the remaining five crayfish were fixed in Bouin’s solution (Phygene, Fuzhou, China) and stored at 4 °C for histological sectioning.

### 2.3. Hepatopancreatic Injury Markers Detection

The hepatopancreatic tissue was homogenized in PBS using a tissue homogenizer at a specified ratio, followed by centrifugation at 4000 rpm and 4 °C for 15 min. The supernatant was collected in centrifuge tubes for further analysis. Hemolymph was directly sampled for assays. Commercial kits (Nanjing Jiancheng Bioengineering Institute, Nanjing, China) were utilized to measure the activities of glutamic-oxaloacetic transaminase (GOT) and glutamic-pyruvic transaminase (GPT) at an absorbance of 510 nm. Protein concentrations were determined using a commercial protein assay kit, with all results normalized to the protein content.

### 2.4. Hepatopancreatic Oxidative Stress Markers Detection

The procedure for hepatopancreatic tissue homogenization was identical to that described in Section 2.3. Assays to measure oxidative stress markers used commercial kits (Nanjing Jiancheng Bioengineering Institute, China) for determining malondialdehyde (MDA) content and superoxide dismutase (SOD) activity at absorbance wavelengths of 532 nm and 450 nm, respectively. Protein concentrations were assessed with a commercial kit, and the results were normalized to protein content to ensure accuracy.

### 2.5. Histological Examination of Hepatopancreas

Hepatopancreatic tissues fixed in Bouin’s solution underwent dehydration through a graded series of ethanol and were cleared with xylene. These prepared tissues were then embedded in paraffin and sectioned at an 8 μm thickness using a Leica paraffin microtome (Leica, Berlin, Germany). The subsequent steps involved the deparaffinization and rehydration of the sections using a graded ethanol series. The sections were stained with hematoxylin for 5–8 min, differentiated with 1% hydrochloric acid in alcohol, and rinsed under running water. Eosin staining was conducted for 1–3 min, after which the sections were dehydrated through a graded ethanol series and mounted with neutral resin. Observations and photographic documentation of the slides were performed using an Olympus microscope (Olympus, Tokyo, Japan).

### 2.6. Hepatopancreatic Fibrosis Detection

Following the histological preparation described in Section 2.5, hepatopancreatic fibrosis was assessed using a modified Sirius Red staining protocol (Solarbio, Beijing, China). Tissue sections were stained with iron hematoxylin for 8 min, followed by rinsing under tap water for 10 min. Staining with Sirius Red solution proceeded for 15 min. After staining, the sections were dehydrated through graded ethanol and mounted with neutral resin. Microscopic observation and photography were conducted using an Olympus microscope (Olympus, Japan).

### 2.7. Metabolomic Analysis

The thawed samples were homogenized for 20 s at 30 Hz using a tissue grinder. A 400 μL solution containing internal standards (methanol:water, 7:3 *v*/*v*) was added to 20 mg of the homogenized tissue. The samples were incubated on ice for 15 min before centrifugation at 12,000 rpm and 4 °C for 10 min. A 300 μL aliquot of the supernatant was collected and stored at −20 °C for 30 min, followed by a second centrifugation under the same conditions for 3 min. The supernatant was then processed for LC-MS/MS analysis using a UHPLC-Q Exactive HF-X system (Thermo Fisher Scientific, Waltham, MA, USA) to conduct ultrahigh-performance liquid chromatography coupled with Fourier-transform mass spectrometry. Data acquisition and processing included the conversion of raw data files to the mzXML format using the ProteoWizard software (v3.0.19254), peak extraction, alignment, and retention time correction using the XCMS program. The peak areas were normalized using the “SVR” method, and metabolites detected in fewer than 50% of samples were excluded. The identified metabolites were annotated using in-house, public, AI databases, and metDNA methods. Orthogonal partial least squares discriminant analysis (OPLS-DA) was conducted using the OPLSR. Anal function in the MetaboAnalystR package of the R software (Version 2.0.0), identifying differentially expressed metabolites (DEMs) based on variable importance in projection (VIP) scores greater than 1 and *p*-values less than 0.05. The top significantly enriched metabolic pathways were identified using the KEGG database and were visualized along with a volcano plot of the DEMs using the Origin 2023 software.

### 2.8. Quantitative Real-Time PCR (qPCR)

The RNA was isolated from homogenized hepatopancreatic tissue using Trizol reagent through a single-step method. RNA integrity was confirmed with an OD 260/280 ratio between 1.8 and 2.2. Reverse transcription was performed using the HiScript II First Strand cDNA Synthesis Kit (+gDNA wiper) (Vazyme Biotech Co., Ltd., Nanjing, China). qPCR analyzed the expression levels of CASP2, NDUFA7, lysozyme, and CYTB, with 18S RNA serving as the internal reference gene. The gene primer sequences used in this study are shown in Table 1. Relative expression changes were quantified using the 2^−ΔΔCq^ method [26].

### 2.9. Data Analysis

All data are presented as the mean ± standard deviation (SD). Statistical analyses were performed using SPSS version 25.0. The Shapiro–Wilk test was used to assess normal distribution, and the Levene’s test was used to assess the homogeneity of variances. Data that did not meet these assumptions were transformed logarithmically prior to analysis by one-way ANOVA. A *p*-value of less than 0.05 was considered statistically significant. Graphical representations, excluding those for transcriptomic and metabolomic data, were generated using Prism 9.0 (GraphPad, Boston, MA, USA).

## 3. Results

### 3.1. Analysis of Hepatopancreatic Injury Markers

**Figure 1:**
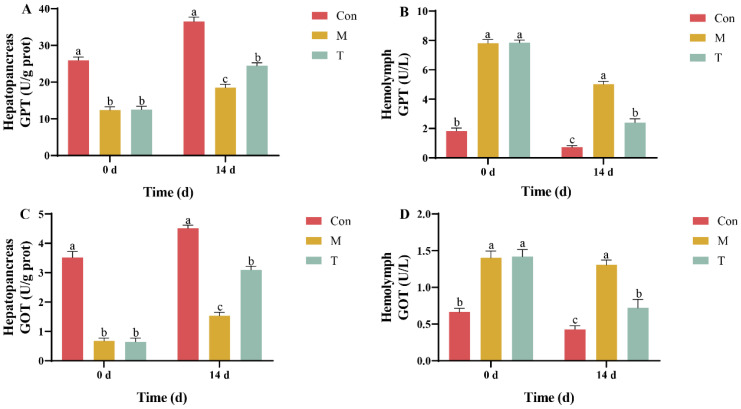
The GOT and GPT activity in the *P. clarkii* hepatopancreas and hemolymph in the Con, M, and T groups. (**A**) Hepatopancreas GPT; (**B**) hemolymph GPT; (**C**) hepatopancreas GOT; (**D**) hemolymph GOT. There is a significant difference between the two groups without the same letter at the *p* < 0.05 level. Data were given as the mean ± SD (*n* = 3).

Initial assessments revealed that glutamic-pyruvic transaminase (GPT) activity in the hepatopancreas was significantly reduced in both the liver injury model group (M group) and the treatment group (T group) compared to the control group (Con group), with no significant initial differences between the M and T groups. By the 14th day of the experiment, the GPT activity in the T group had significantly increased compared to the M group, indicating a recovery trend (Figure 1A). Throughout the experiment, the GPT activity in the hemolymph of the T group exhibited a declining trend and was significantly lower than that in the M group by day 14 (Figure 1B). In terms of glutamic-oxaloacetic transaminase (GOT) activity, there was no significant difference between the T and M groups at the experiment’s start. However, by day 14, the GOT activity in the T group had significantly increased compared to the M group, though it remained lower than that observed in the Con group (Figure 1C). Furthermore, the GOT activity in the hemolymph of the T group showed a significant decrease by day 14 (Figure 1D).

### 3.2. Analysis of Oxidative Stress Markers in the Hepatopancreas

**Figure 2:**
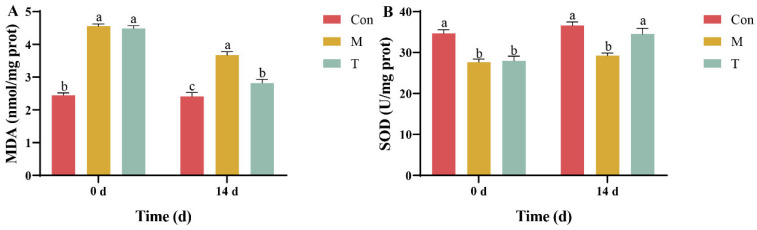
MDA content and SOD activity in the *P. clarkii* hepatopancreas in the Con, M, and T groups. (**A**) MDA; (**B**) SOD. There is a significant difference between the two groups without the same letter at the *p* < 0.05 level. Data were given as the mean ± SD (*n* = 3).

At the experiment’s outset, both the M and T groups exhibited marked oxidative damage as evidenced by the malondialdehyde (MDA) content in the hepatopancreas, which was 1.86-fold and 1.84-fold higher than that of the Con group, respectively. By the end of the experiment, the MDA content in the T group had significantly decreased to only 1.17-fold of the Con group, demonstrating the efficacy of astaxanthin treatment in reducing oxidative stress (Figure 2A). Additionally, the recovery of superoxide dismutase (SOD) activity in the T group by day 14 was notable, with levels comparable to those of the Con group, indicating no significant difference (Figure 2B). The results highlight that astaxanthin treatment effectively mitigated the TAA-induced elevation in MDA levels and the reduction in SOD activity in the hepatopancreas of *P. clarkii* throughout the study period.

### 3.3. Histological Examination

**Figure 3:**
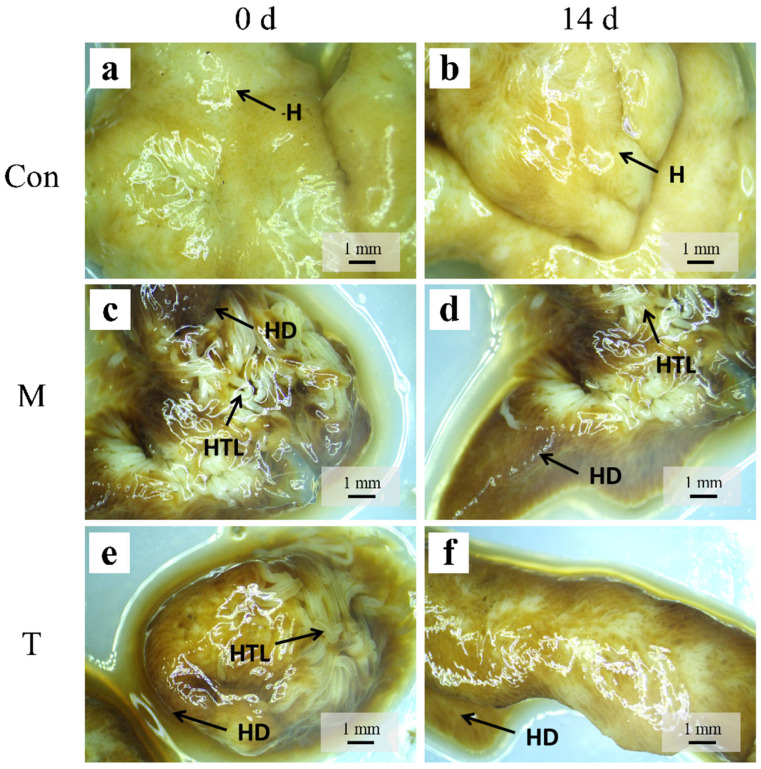
A stereomicroscopic observation of the *P. clarkii* hepatopancreas. (**a**,**b**): Con; (**c**,**d**): M; (**e**,**f**): T. H: Hepatopancreas; HD: hepatopancreas dark; HTL: hepatic tubules LOOSE.

Histological assessments were conducted using a stereomicroscope to observe the structural changes in the hepatopancreas of crayfish across different experimental groups. In the control group (Con group), the hepatopancreas exhibited a light yellow hue, characterized by a dense structural arrangement of finely detailed hepatic tubules that were intact and uniformly distributed (Figure 3a,b). This normal morphology contrasts starkly with observations following acute liver injury induction by thioacetamide (TAA). Post-TAA exposure, the hepatopancreas in both the model (M group) and treatment (T group) groups displayed marked pathological changes. Initially, there was a noticeable darkening of the tissue color, and the hepatic tubules appeared loose and coarsely structured (Figure 3c,e). By day 14, the M group showed further deterioration; the hepatic tubules were increasingly disorganized, and the color deepened to a more pronounced dark brown (Figure 3d). Conversely, the T group exhibited significant histological recovery; the dark brown discoloration of the hepatopancreas lightened, and the hepatic tubules were more tightly organized, suggesting the effective mitigation of TAA-induced damage (Figure 3f).

**Figure 4:**
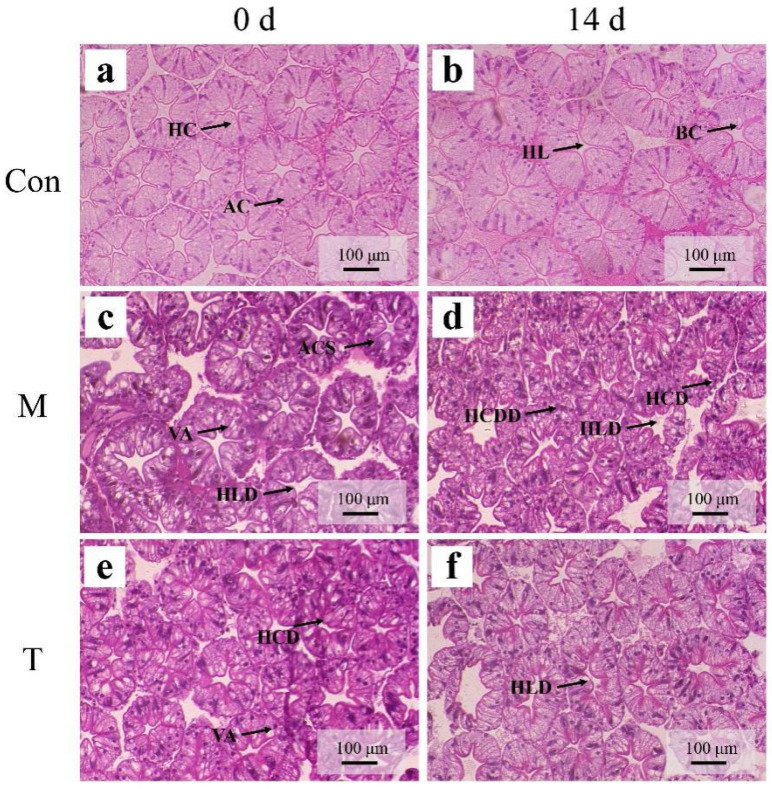
HE staining diagrams of the *P. clarkii* hepatopancreas. (**a**,**b**): Con; (**c**,**d**): M; (**e**,**f**): T. HC: Hepatic canaliculi; HL: hepatic lumen; BC: bulliform cell; AC: absorptive cell; HLD: hepatic lumen deformation; HCD: hepatic canaliculi damage; HCDD: hepatic canaliculi distribution disorder; VA: vacuolar anomaly; ACS: absorptive cell swelling.

Throughout the experiment, the control group (Con group) displayed well-preserved hepatopancreatic tubule structures. These structures featured stellate-shaped lumens, plump cells, and a uniform distribution, indicative of healthy tissue morphology (Figure 4a,b). At the onset of the experiment, both the liver injury model group (M group) and the treatment group (T group) demonstrated significant morphological changes. These included deformed hepatic lumens and some hepatopancreatic tubules showing signs of damage. Notably, the absorptive cells (R cells) appeared swollen, and the bubble cells (B cells) were characterized by the presence of small vacuoles, suggesting initial injury responses (Figure 4c,e). By day 14, the condition of the hepatopancreas in the M group had worsened considerably. This was evidenced by severely swollen R cells, tubules that were disrupted and adhered, compressing each other, and irregularly shaped lumens. Additionally, the B cells were filled with numerous small vacuoles, indicating progressive hepatopancreatic damage (Figure 4d). Conversely, the T group showed marked improvements; there was a significant reduction in the swelling of the R cells and a decrease in the number of small vacuoles within the B cells, illustrating a substantial alleviation of the induced damage (Figure 4f).

### 3.4. Sirius Red Staining

**Figure 5:**
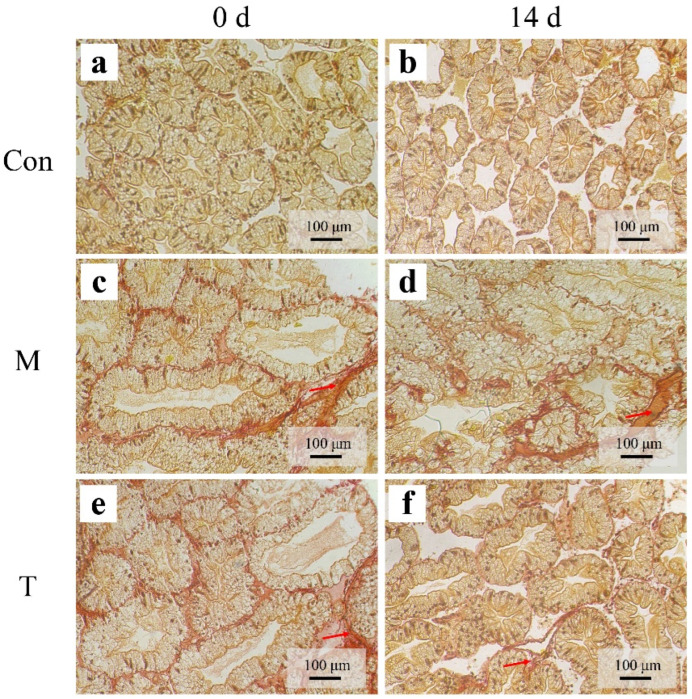
Sirius Red staining diagrams of the *P. clarkii* hepatopancreas. (**a**,**b**): Con; (**c**,**d**): M; (**e**,**f**): T. The red arrows indicate fibrotic tissues.

Sirius Red staining, which specifically identifies collagen fibers indicative of fibrosis, was employed to assess the extent of fibrotic changes in the hepatopancreatic tissues. Throughout the duration of the experiment, the control group (Con group) exhibited no signs of fibrosis, maintaining normal tissue architecture (Figure 5a,b). Initially, mild fibrosis was noted in both the liver injury model group (M group) and the treatment group (T group), characterized by small amounts of fibrotic tissue between the hepatopancreatic tubules as highlighted by the red arrows (Figure 5c,e). By day 14, there was a significant exacerbation of fibrosis in the M group (Figure 5d), whereas the T group displayed a notable reduction in fibrotic tissue, indicating an improvement in the tissue condition (Figure 5f).

### 3.5. Metabolomic Analysis Results

**Figure 6:**
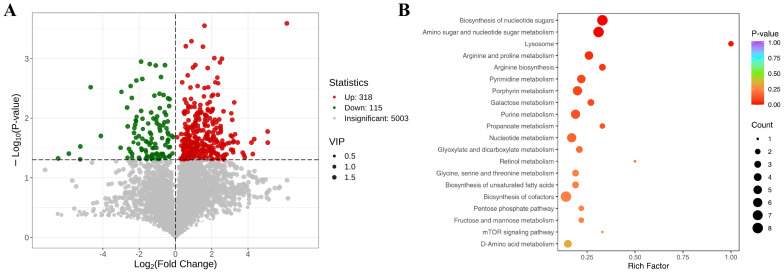
Metabolomic analysis results of M and T groups in 14 d. (**A**) DEMs volcano map; (**B**) analysis diagram of KEGG enrichment in metabolome.

Metabolomic analysis performed on day 14 revealed a total of 987 differentially expressed metabolites (DEMs) between the M group and the T group. This included 599 metabolites that were significantly upregulated and 388 that were significantly downregulated (Figure 6A). The twenty most significant items for metabolomic analysis are shown in Table 2. Pathway enrichment analysis of these DEMs indicated significant alterations in several key metabolic pathways, including the biosynthesis of nucleotide sugars, lysosome function, arginine and proline metabolism, pyrimidine metabolism, porphyrin metabolism, galactose metabolism, and fructose and mannose metabolism (Figure 6B). These findings highlight the profound metabolic shifts that occurred as a response to the experimental conditions and treatment.

### 3.6. qPCR Analysis

**Figure 7:**
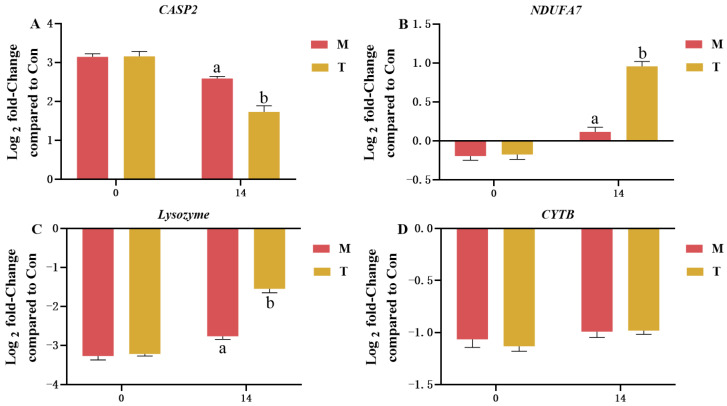
The expression of different genes in the M and T groups. (**A**) *CASP2*; (**B**) *NDUFA7*; (**C**) *Lysozyme*; (**D**) *CYTB***.** The x-axis values 0 and 14 represent 14 days after the experimental treatment. There is a significant difference between the two groups without the same letter at the *p* < 0.05 level. There is no significant difference between groups without a letter. Data were given as the mean ± SD (*n* = 3).

qPCR was used to analyze gene expression differences in the hepatopancreas of the M group and T group at the start (day 0) and end (day 14) of the experiment. The initial results indicated no significant differences in gene expression between the groups at the outset of the experiment. However, by day 14, the astaxanthin treatment had led to a significant downregulation of the *CASP2* and *NDUFA7* genes, while significantly upregulating lysozyme expression in the T group. There was no significant change in *CYTB* gene expression between the two groups (Figure 7). These results demonstrate the regulatory effect of astaxanthin on key genes related to cell apoptosis, energy metabolism, and immune response, underscoring its potential therapeutic impact in the hepatopancreas of *P. clarkii*.

## 4. Discussion

TAA is a well-documented hepatotoxic agent that induces liver injury by impairing protein synthesis and modifying intracellular enzyme metabolism through interactions with the hepatic cytochrome P450 enzyme system. This property has led to its extensive application in liver pathology studies across various animal models, facilitating insights into hepatic injury mechanisms and potential therapeutic interventions [19]. In line with these applications, our laboratory successfully utilized TAA to establish a hepatopancreatic injury model in crayfish, providing a platform for investigating the reparative effects of the AST-product on hepatopancreatic tissues.

GPT and GOT are critical aminotransferases located in the mitochondria of the hepatopancreas, serving as reliable indicators of tissue damage and dysfunction [20]. Typically, under normal physiological conditions, these enzymes are minimally present in the hemolymph. Consequently, observed fluctuations in their activity, particularly decreases in the hepatopancreas coupled with increases in the hemolymph, are indicative of hepatopancreatic injury [21]. In this context, the AST-product has demonstrated significant efficacy in reducing the TAA-induced elevations of GPT and GOT activities, aligning with findings from other studies where synthetic astaxanthin diminished these enzyme activities in *Penaeus monodon*, thereby safeguarding hepatopancreatic health [22]. These changes are also reflected histologically, where AST-product-treated crayfish showed reduced tissue disorganization and cellular swelling, indicating improved tissue recovery.

Furthermore, SOD and MDA are pivotal oxidative stress markers. SOD plays a crucial role in catalyzing the conversion of superoxide radicals into hydrogen peroxide, which is subsequently broken down by catalase (CAT) and glutathione peroxidase (GPX) into less harmful compounds, thus mitigating oxidative stress [23,24]. An increase in MDA content typically indicates heightened oxidative stress and potential tissue damage [25]. Lipid peroxidation (with MDA as a product) is a primary pathway by which ROS cause tissue damage and is commonly regarded as a marker of oxidative damage, which is used to assess the health status of organisms [26]. An increase in MDA content indicates potential oxidative stress damage in organisms. Notably, in our study, the AST-product significantly enhanced SOD activity while reducing MDA levels, suggesting the effective amelioration of oxidative stress induced by TAA. This observation is supported by similar findings in other studies, where astaxanthin improved oxidative stress markers in *Exopalaemon carinicauda* [27]. These biochemical results were in strong agreement with histopathological findings, which showed improved hepatopancreatic architecture, including more defined tubule lumens and reduced vacuolization in the AST-treated groups.

In addition to histological and biochemical improvements, our metabolomic data revealed systemic metabolic adjustments associated with AST-product intervention. Significant changes were observed in multiple metabolic pathways, including those related to amino acid, energy, and nucleotide metabolism, suggesting a comprehensive biochemical response to AST-product treatment. Notably, alterations in arginine and proline metabolism may bolster the production of endogenous hydrogen peroxide, enhancing cellular antioxidant defenses [28,29,30]. Additionally, the modulation of porphyrin metabolism could help regulate heme levels, thereby mitigating oxidative stress and preventing cellular apoptosis [31]. These metabolic adjustments are critical for maintaining genomic stability and fulfilling the hepatopancreas’s energy demands [32,33]. The improvement of galactose, fructose, and mannose metabolism observed in the treatment group suggests an increased flow of hexose phosphates into energy-producing pathways or glycogen synthesis [34,35,36]. This is supported by reduced hepatopancreatic vacuolization and improved tissue compactness, which likely reflect restored energy balance and membrane stabilization.

Studies have shown that the upregulation of *CASP2* gene expression in the hepatopancreas induces apoptosis [37,38]. From a genetic perspective, the AST-product’s regulation of key genes such as *CASP2*, *NDUFA7*, and *lysozyme* highlights its potential to influence cellular apoptosis, energy production, and innate immune responses, respectively. The suppression of *CASP2* expression mitigates apoptosis, while the upregulation of *NDUFA7* enhances mitochondrial function, improving energy efficiency within cells [39]. Moreover, the induction of lysozyme expression bolsters the organism’s defense against pathogens, playing a vital role in the innate immune system [40,41]. These molecular signatures corroborate the phenotypic improvements observed in antioxidant markers, metabolomic profiles, and histopathology, providing a multidimensional understanding of the AST-product’s hepatoprotective effects.

## 5. Conclusions

This study demonstrated that the AST-product exerts significant protective effects against TAA-induced hepatopancreatic injury in *P. clarkii*. The therapeutic efficacy of the AST-product was achieved through the regulation of multiple metabolic pathways, particularly those involved in amino acid, energy, and nucleotide metabolism. This metabolic modulation effectively alleviated oxidative stress, preserved the hepatocyte structure, and inhibited the progression of hepatopancreatic fibrosis. Moreover, the AST-product enhanced the innate immune capacity of *P. clarkii* by modulating the expression of key genes, including *CASP2*, *NDUFA7*, and *lysozyme*, which are associated with apoptosis, mitochondrial function, and antimicrobial defense. Collectively, these findings highlight the AST-product as a promising functional additive for improving hepatopancreatic health and provide new theoretical and practical foundations for disease prevention and sustainable aquaculture practices.

## Figures and Tables

**Table 1:** Primers for qPCR.

Gene Name	Accession Number	Primer Name	Primer Sequence 5′ to 3′	Tm (°C)	Product (bp)
*18S*	XM_045748331	F	TCGGCATGGCATGGTTAA	58.6	203
		R	ACGGCAAGAGCCTTGGAT	57.6	
*CASP2*	XM_045739219	F	CCCTTGGCATCTTTACCTTACA	58.0	186
		R	AATACTAGGGAAGATCAGAGCAGG	59.1	
*NDUFA7*	XM_045751651	F	TGCGTCAAGCAGACATTA	56.2	174
		R	CAGATAACAGTTTGGTGGG	56.2	
*Lysozyme*	XM_069326239	F	GAGGATGTGGTCGTGGGTGA	60.5	271
		R	ATTGGTCGTTCTAATGCCGC	58.9	
*CYTB*	MN982313	F	AAGTTGAAATAAGGGTGAAAGG	55.3	190
		R	GGATTTGAGGTGGCTTCG	55.8	

**Table 2:** The twenty most significant items for metabolomic analysis.

Index	Compounds	*p*-Value	Type
MW0141633	17-phenyl trinor PGF2 diethyl amide	2.88 × 10^−6^	down
MW0141691	1-Amino-1-deoxy-scyllo-inositol 4-phosphate	9.81 × 10^−6^	down
MW0015058	(R)-Sulcatol	2.72 × 10^−5^	down
MW0112744	Acetamidopropanal	3.44 × 10^−5^	up
MW0159233	Val-Val-Asn-Trp-Asp	1.19 × 10^−4^	up
MW0015307	8-Deoxy-11,13-dihydroxygrosheimin	2.13 × 10^−4^	up
MW0002106	2,3′,4,6-Tetrahydroxybenzophenone	2.18 × 10^−4^	down
MW0145457	Arg-Phe-Ala	2.27 × 10^−4^	up
FDATN00822	Propacetamol hydrochloride	2.63 × 10^−4^	up
MW0052901	Desferal-iron(III)	2.71 × 10^−4^	down

## Data Availability

The data that support the findings of this study are available from the corresponding author upon reasonable request.
